# The impact of surge capacity enhancement training for nursing managers on hospital disaster preparedness and response: an action research study

**DOI:** 10.1186/s12873-024-00930-1

**Published:** 2024-08-26

**Authors:** Alireza Shafiei, Narges Arsalani, Mehdi Beyrami Jam, Hamid Reza Khankeh

**Affiliations:** 1 Department of Nursing, University of Welfare and Rehabilitation Sciences, Tehran, Iran; 2 Iranian Research Center on Aging, Department of Nursing, University of Social Welfare and Rehabilitation Sciences, Tehran, Iran; 3 Department of Pre-Hospital Medical Emergencies, School of Paramedical, Qazvin University of Medical Sciences, Qazvin, Iran; 4 Health in Emergency and Disaster Research Center, University of Social Welfare and Rehabilitation Sciences, Tehran, Iran

**Keywords:** Hospital, Disaster preparedness, Surge capacity, Action research, Nurse

## Abstract

**Introduction:**

Hospitals as the main providers of healthcare services play an essential role in the management of disasters and emergencies. Nurses are one of the important and influential elements in increasing the surge capacity of hospitals. Accordingly, the present study aimed to assess the effect of surge capacity enhancement training for nursing managers on hospital disaster preparedness and response.

**Methods:**

All nursing managers employed at Motahari Hospital in Tehran took part in this interventional pre- and post-test action research study. Ultimately, a total of 20 nursing managers were chosen through a census method and underwent training in hospital capacity fluctuations. The Iranian version of the “Hospital Emergency Response Checklist” was used to measure hospital disaster preparedness and response before and after the intervention.

**Results:**

The overall hospital disaster preparedness and response score was 184 (medium level) before the intervention and 216 (high level) after the intervention. The intervention was effective in improving the dimensions of hospital disaster preparedness, including “command and control”, “triage”, “human resources”, “communication”, “surge capacity”, “logistics and supply”, “safety and security”, and “recovery”, but had not much impact on the “continuity of essential services” component.

**Conclusion:**

The research demonstrated that enhancing the disaster preparedness of hospitals can be achieved by training nursing managers using an action research approach. Encouraging their active participation in identifying deficiencies, problems, and weaknesses related to surge capacity, and promoting the adoption and implementation of suitable strategies, can enhance overall hospital disaster preparedness.

## Introduction

Hospitals, as the main providers of healthcare services, play an essential role in managing and reducing the suffering of injured people in emergencies and disasters [26]. Most of the definitive, life-saving and emergency care for injured people are carried out in hospitals. Therefore, the preparedness of hospitals is essential in moderating and decreasing the negative health consequences of disasters [29]. From an international perspective, the Sendai Framework for Disaster Risk Reduction 2015–2030 and World Health Organization (WHO), highlights the need for disaster preparedness and risk reduction measures in hospitals [30, 31]. Based on WHO, the preparedness and well-trained hospital personnel is the main factor in minimizing the casualties and damages resulting from disasters. Therefore, assessing and improving hospitals’ capacity and preparedness for disasters is a crucial first step toward effective disaster response and achieving the objectives outlined in the Sendai Framework 2015–2030 [30, 32].

In Iran, efforts to enhance hospitals’ disaster preparedness began in the winter of 2009 with the creation of the National Hospital Disaster Preparedness Plan (NHDPP) by the Health Research Center on Disasters at the University of Social Welfare and Rehabilitation Sciences. This initiative, serving as a national guideline, received backing from the Secretariat of the Disaster Health Working Group in the Ministry of Health and was communicated to all hospitals across the country [1]. Furthermore, in the third phase of Iran’s hospital accreditation program, criteria for disaster risk management were added in the form of seven standards and thirty-seven measurements, directly addressing the hospital’s preparedness and response to emergencies and disasters [2].

To effectively address disasters, a hospital needs a thorough preparedness strategy, necessary tools, equipment, sufficient space, skilled staff, and, in essence, enough surge capacity [33]. Surge capacity refers to the ability to acquire additional resources during a disaster or emergency. It is the ability to provide quickly the usual functions beyond the increased demand for experienced staff, medical care, and social health services. Surge capacity has three core components including staff, stuff, and structures [3].

Nurses are one of the major groups of healthcare providers in hospitals(staff) [4]. They have the most contact with patients and provide the most care [5]. Along with other disaster management teams, they also play crucial roles in planning, education and training, response, and recovery for hospital disaster preparedness [6, 7].

Experiences have shown that training and exercises before the occurrence of disasters can significantly increase the ability of people to face critical situations such as natural disasters [4, 6]. Therefore, providing effective disaster training for nurses has a crucial role in increasing hospital preparedness and capacity for response to disasters. Previous studies have demonstrated inadequate training for nurses on preparedness and response to emergencies and disasters [2, 4–6]. Moreover, despite numerous investigations assessing the preparedness of Iranian hospitals for disasters [8–10], to the best of our knowledge, only a limited number of interventional studies have explored the impact of disaster training for nurses on enhancing hospital disaster preparedness in Iran. Hence, recognizing the crucial contributions of nurses to the development of hospital capacity, this research aimed to examine the effects of training of surge capacity enhancement for the nursing managers on the emergency and disaster preparedness of Motahari Hospital in Iran.

## Methods

### Study design and settings

The current investigation utilized a pretest-posttest interventional design, conducted at Shahid Motahari Burn Hospital, affiliated with Iran University of Medical Sciences in Tehran, Iran. This hospital is the first and only main and specialized center providing medical services to burn patients in the center of the country and plays an essential role in the management of the injured during disasters and emergencies, especially fires.

### Population and sampling

Aligned with the study’s goals, we employed a census sampling method to select all nursing managers at Shahid Motahari Hospital in Tehran. The eligibility criteria encompassed individuals within the nursing profession, such as nursing managers, supervisors, and head nurses, who held a minimum of a bachelor’s degree and possessed a minimum of one year of managerial experience. Those who expressed unwillingness to participate in the study were excluded.

### Instrument

The data was collected using the Persian version of the Hospital Emergency Response Checklist developed by Khankeh et al. (2013) [34]. The checklist was used to estimate the current state of preparedness of hospitals and healthcare centers. The original version of this tool was formulated by the World Health Organization [35]. The checklist measures 9 key components including command and control (7 items), triage (10 items), human resources (15 items), communication (9 items), surge capacity (13 items), logistics and supply management (10 items), safety and security (10 items), continuity of essential services (8 items) and post-disaster recovery (8 items). The reliability and validity of the Persian version of the tool have been confirmed by Karimian et al. (2013) [14]. They confirmed the validity of the tool (CVI = 0.86) and its reliability with Cronbach’s alpha of 0.83. The items in the checklist are rated on a 3-point scale (1 = due for review, 2 = in progress, and 3 = completed).

Moreover, the hospital surge capacity guideline was used to examine the current situation, weaknesses, problems, and target actions and develop a hospital surge capacity training program. This guidance was formulated by the Health in Emergency and Disaster Research Center at the University of Social Welfare and Rehabilitation Sciences and approved and disseminated by the Iranian Ministry of Health [34].

### Intervention

This intervention study adopted a participatory action research approach as the participants were involved in problem identification and intervention to improve the process. Research in action is a type of study used by people to change unfavorable situations into relatively favorable situations and finally improve procedures in their workplace [11]. Action research is a type of study that attempts to learn and understand purposeful interventions meant to bring about desired changes in the organizational environment [12]. Action research simultaneously promotes problem-solving and expands scientific knowledge, as well as strengthens the skills of research participants [13].

In general, in action research, participants are involved in all stages of the research, from identifying the problem and collecting the data to planning, implementation, and evaluation. The engagement of participants in all stages of the research will encourage their participation in the research procedure and make them interested in the research topic [7].

This study adopted Streubert Speziale and Carpenter’s five-step action research method [7]. These steps include (1) defining the problem (explaining the current situation), (2) collecting, analyzing, and interpreting data, (3) planning, (4) implementing, and (5) evaluating. In this research, nurses actively engaged in elucidating the issue, gathering and analyzing data related to hospital surge capacity, devising and executing capacity-enhancing strategies based on their training, and assessing these measures to enhance hospital disaster preparedness and response.

To collect the data, the required permits were obtained from the hospital managers and officials. Besides, some instructions about the research procedure and data gathering were provided in a briefing session for the participants. The researcher and the participants made the required arrangements and plans for conducting the training intervention. In the next step, the items on the instruments (the Hospital Emergency Response Checklist) were completed by the participants(pre-test). When completing the checklist, the officials and managers of the hospital were also interviewed to better identify the problems and challenges related to the surge capacity. After that, topics and concepts related to increasing surge capacity and hospital disaster preparedness were taught to the participants during a two-day workshop, and they did round table exercises. Following the National Hospital Emergency Preparedness and Response Instructions [1], the content of the workshop included hospital risk and hazard assessment, incident command system, early warning system, response plan, and enhancing hospital capacity in response to emergencies and disasters with emphasis on solving problems and weaknesses identified in the pre-intervention stage. After completing the training workshop, the participants were given a six-month opportunity to carry out interventions and transfer the training to other staff and nurses. During this period, the participants and other members of the disaster risk management committee attended meetings held every two weeks. In these meetings, the necessary actions for the next two weeks were set, and the officials to manage each action were specified. In addition, in each meeting, the extent to which the goals of the previous meeting were achieved and the reasons for not fulfilling them were discussed. Finally, the items in the Hospital Emergency Response Checklist were completed for the second time (post-test) and the collected data was analyzed.

### Ethical considerations

To comply with ethical protocols, this research project was approved with the code of ethics of the Ethics Committee of the University of Rehabilitation Sciences and Social Health. Moreover, informed consent was obtained from all the participants. The participants completed the checklists anonymously and, they were assured that their participation was voluntary and had no impact on their evaluation procedure.

## Results

The participants in this study were 20 nursing managers and supervisors at Motahari Burn Hospital in Iran. The study participants had an average age of 38 years (30 to 52 years old) and an average work experience of 16 years (4 to 25 years). Most of the participants were female (15 persons), married (18 persons), had a bachelor’s degree (12 persons), and had served in managerial positions (9 persons). Table No. 1 Shows other demographic characteristics of the participants. The surge capacity enhancement strategies that were recognized and put into practice by the participants throughout the study(6 months) included: 1- Executing a memorandum with retired personnel and reactivating them when necessary, Executing a memorandum with the Iran University of Medical Sciences to hire students if needed, drafting instructions for requesting staff from the relevant authorities such as the Emergency Operations Center (EOC) of the Ministry of Health, in the realm of enhancing “staff” capacity. 2- Preparing and reserving medications and essential equipment for a minimum duration of 72 h, signing a memorandum with other hospitals and nearby health centers to provide equipment in emergencies, and also creating more water storage volume to be used in emergencies and disasters, in the realm of enhancing “stuff” capacity. 3- Identifying suitable non-clinical and clinical spaces in the Motahhari Hospital to place beds and admit patients during disasters and emergencies, concluding an agreement with a school near the hospital to provide physical space for the hospital, creating a new rehabilitation department in the hospital, enlarging the space of the emergency department in the realm of increasing “space” capacity. And, 4- developing plans and instructions necessary to manage the risk of emergencies and disasters, doing training and practice in the hospital, in the realm of enhancing “system” capacity. The data showed that hospital disaster preparedness was at an average level (184) before the intervention and reached the optimal level (216) after the intervention. Also, the results also demonstrated that, except for “continuity of essential services”, the intervention improved the hospital’s disaster preparedness score across all dimensions. Most notably, the intervention enhanced “surge capacity” by 10 units and “staff” by 6 units. For detailed information on the intervention’s effects on hospital preparedness dimensions, please refer to Table No. 2.

**Table 1:** Demographic and professional information on nursing managers

Variables	Categories	Frequency	Percentage
Gender	Female	15	75
	Male	5	25
Work experience as a manager	Clinical nursing manager	9	45
	Active in Hospital disaster committee	11	55
Educational degree	Bachelor’s degree	12	60
	Master’s degree	7	35
	Ph.D	1	5

**Table 2:** The hospital disaster preparedness score before and after the intervention

Key research tool components	Preparedness score		
	Before intervention	After intervention	Score difference
Command and control	13	18	5
Communication	17	20	3
Safety and Security	21	22	1
Triage	17	21	4
Surge capacity	25	35	10
Continuity of essential services	22	22	0
Human resources	27	33	6
logistics and supply	27	28	1
Recovery	15	17	2
Total disaster preparedness	184	216	32

## Discussion

This study aimed to examine how providing action research training to nursing managers enhances surge capacity and contributes to improving hospital disaster preparedness. Many hospitals may face numerous challenges due to inadequate preparedness in the face of disasters and the increased demand for healthcare services [36, 37]. The results of this study indicated that implementing the surge capacity enhancement intervention for nursing managers and officials led to a 32-unit improvement in disaster preparedness at Motahari Hospital. This improvement was expected because surge capacity is one of the most important components of hospital disaster preparedness and response.

Regarding the impact of the intervention on enhancing hospital disaster preparedness, various studies have been conducted in Iran, each employing distinct approaches to bolster preparedness.

In a study conducted by Karimiyan et al. (2013), it was found that hospital preparedness training aligned with the national plan significantly enhanced the hospital’s preparedness to address emergencies and disasters [14]. Delshad et al. (2015) showed early warning system training improved the preparedness of Motahari Hospital in emergencies and disasters [15]. Also, Salawati et al. (2014) in another study, examined the effect of teaching and applying non-structural hospital safety principles for nurses on the preparedness of medical departments of several private and public hospitals in Tehran during disasters [16]. The findings indicated that the safety score of two non-structural and functional parts of the hospital safety index increased after the intervention. The authors concluded that teaching and applying non-structural safety principles to nurses improves hospital safety and preparedness [16].

Like numerous other hospitals in Iran [17–19], Motahari Hospital’s disaster preparedness status was assessed as moderate before the intervention. Nevertheless, some studies have indicated inadequacies in the preparedness level of the examined hospital. For example, both the investigation conducted by Hekmatkhah et al. [20] and that of Ojaghi et al. [21] revealed insufficient preparedness in the hospitals under examination.

The current study demonstrated that enhancing the hospital’s response capacity and hospital’s disaster preparedness across various components can be achieved through capacity-building training for nursing managers through action research. The greatest effect of the intervention in this study was on “surge capacity” and the “human resource” dimension(staff). This outcome can be primarily attributed to instructing the hospital surge capacity-building principles for participants in the training workshop. Additionally, due to steps were taken to augment capacity in terms of “human resources”, “medication, and equipment”. Two studies conducted in Iran have identified a shortage of human resources and equipment as a primary factor contributing to the limited preparedness of hospitals in dealing with disasters [22, 23]. In this research, the re-employment of retired employees and the use of university students were among the most important strategies that were adopted to increase the hospital capacity and preparedness in the human resource dimension. Similarly, Dowlati et al. (2021) reported that the preparation of a list of employers from other hospitals and medical centers, including clinics and health students, is one of the most important strategies to increase the capacity of hospital staff to respond to chemical, biological, and nuclear hazards and disasters [38].

The results of this study show that the intervention improved the hospital preparedness scores in the “triage” and “command and control” dimensions. In this context, the educational intervention on triage by Rahmati and colleagues enhances the preparedness of the emergency department, as highlighted in their study [24]. Also, Delshad et al. conducted a study where actions such as designating an external location for triage and formulating a strategy for the postponement of elective surgeries contributed to an improvement in the hospital preparedness score [15].

The results of this study emphasize that enhancing hospital preparedness can be achieved through conducting a needs assessment, recognizing gaps within the organization as identified by study participants, and effectively communicating and raising awareness among hospital managers. In this context, Karimian et al. (2013) underscored the importance of providing additional training for officials, managers, and hospital staff concerning emergency preparedness and response in hospitals [14].

The data in the present study indicated the intervention had a smaller impact on the components of “continuity of essential services”, “logistics and supply”, and “safety and security” compared to other components of hospital preparedness. Perhaps one of the main reasons was the restricted timeframe of the study and limited financial resources to carry out capacity-building and preparedness measures in these dimensions. As stated earlier, measures to increase the surge capacity and improve preparedness were formulated and followed up during the meetings of the emergency and disaster risk committees. Since these meetings were held every two weeks, the 6-month timeframe of the study did not leave an opportunity to carry out measures to improve the mentioned components. Furthermore, the limited financial resources can be considered one of the main reasons for not carrying out the actions planned by the committee. The findings of the “logistics” and “essential services” are consistent with the findings of the study by Ingrassia et al. (2016). This study showed that hospital preparedness in these dimensions was poor [25]. The findings concerning the " logistics and supply” as well as the “countiniuty of essential services “dimensions in this research align with the outcomes observed in Ingrassia et al.‘s (2016) study, highlighting the inadequate preparedness of the hospital in these aspects [25].

### Limitations

The study was constrained by a limited duration of 6 months and insufficient financial resources, restricting the ability to implement further measures to enhance hospital preparedness. Future investigations could overcome these limitations by extending the study period to at least one year and ensuring adequate financial resources. Furthermore, as this study solely assessed the impact of the intervention on the disaster preparedness level of a single hospital, statistical analysis could not be conducted due to the absence of mean and standard deviation data. The alterations were solely presented descriptively.

## Conclusion

This study examined the effect of surge capacity training using an action research plan on disaster preparedness and response at Shahid Motahari Hospital in Tehran. The results showed that surge capacity enhancement training for nursing managers and officials increased their sensitivity to the importance of hospital emergency preparedness and response. Furthermore, their proactive involvement in recognizing capacities, deficiencies, problems, and weaknesses with appropriate tools and taking measures to address them can improve hospital emergency preparedness and response. The findings indicated that senior managers within the hospital can instigate changes through the provision of financial backing and the implementation of mandatory protocols.

## Data Availability

The datasets that were used and/or analyzed during the current study are available from the corresponding author upon reasonable request.
